# Effects of Empagliflozin in Type 2 Diabetes With and Without Chronic Kidney Disease and Nondiabetic Chronic Kidney Disease: Protocol for 3 Crossover Randomized Controlled Trials (SiRENA Project)

**DOI:** 10.2196/56067

**Published:** 2024-05-29

**Authors:** Steffen Flindt Nielsen, Camilla Lundgreen Duus, Niels Henrik Buus, Jesper Nørgaard Bech, Frank Holden Mose

**Affiliations:** 1 University Clinic in Nephrology and Hypertension Gødstrup Hospital and Aarhus University Herning Denmark; 2 Department of Clinical Medicine Aarhus University Aarhus Denmark; 3 Department of Renal Medicine Aarhus University Hospital Aarhus Denmark

**Keywords:** SGLT2i, empagliflozin, renal function, blood flow, DM2, diabetes mellitus type 2, CKD, chronic kidney disease, vascular function, sodium-glucose cotransporter 2 inhibitors, T2DM, type 2 diabetes mellitus, randomized controlled trial, RCT, CVD, placebo, renal, recruitment, Denmark, cardiovascular disease

## Abstract

**Background:**

Sodium-glucose-cotransporter 2 inhibitors (SGLT2is) have revolutionized the treatment of type 2 diabetes mellitus (DM2) and chronic kidney disease (CKD), reducing the risk of cardiovascular and renal end points by up to 40%. The underlying mechanisms are not fully understood.

**Objective:**

The study aims to examine the effects of empagliflozin versus placebo on renal hemodynamics, sodium balance, vascular function, and markers of the innate immune system in patients with DM2, DM2 and CKD, and nondiabetic CKD.

**Methods:**

We conducted 3 double-blind, crossover, randomized controlled trials, each with identical study protocols but different study populations. We included patients with DM2 and preserved kidney function (estimated glomerular filtration rate >60 mL/min/1.73 m^2^), DM2 and CKD, and nondiabetic CKD (both with estimated glomerular filtration rate 20-60 mL/min/1.73 m^2^). Each participant was randomly assigned to 4 weeks of treatment with either 10 mg of empagliflozin once daily or a matching placebo. After a wash-out period of at least 2 weeks, participants were crossed over to the opposite treatment. End points were measured at the end of each treatment period. The primary end point was renal blood flow measured with ^82^Rubidium positron emission tomography–computed tomography (^82^Rb-PET/CT). Secondary end points include glomerular filtration rate measured with ^99m^Technetium-diethylene-triamine-pentaacetate (^99m^Tc-DTPA) clearance, vascular function assessed by forearm venous occlusion strain gauge plethysmography, measurements of the nitric oxide (NO) system, water and sodium excretion, body composition measurements, and markers of the complement immune system.

**Results:**

Recruitment began in April 2021 and was completed in September 2022. Examinations were completed by December 2022. In total, 49 participants completed the project: 16 participants in the DM2 and preserved kidney function study, 17 participants in the DM2 and CKD study, and 16 participants in the nondiabetic CKD study. Data analysis is ongoing. Results are yet to be published.

**Conclusions:**

This paper describes the rationale, design, and methods used in a project consisting of 3 double-blind, crossover, randomized controlled trials examining the effects of empagliflozin versus placebo in patients with DM2 with and without CKD and patients with nondiabetic CKD, respectively.

**Trial Registration:**

EU Clinical Trials Register 2019-004303-12; https://www.clinicaltrialsregister.eu/ctr-search/search?query=2019-004303-12, EU Clinical Trials Register 2019-004447-80; https://www.clinicaltrialsregister.eu/ctr-search/search?query=2019-004447-80, EU Clinical Trials Register 2019-004467-50; https://www.clinicaltrialsregister.eu/ctr-search/search?query=and+2019-004467-50

**International Registered Report Identifier (IRRID):**

DERR1-10.2196/56067

## Introduction

### Overview

The World Health Organization has recently named diabetes the ninth leading cause of death globally [1]. Worldwide, more than 500 million people are living with diabetes. By 2045, this number is expected to rise to more than 780 million [2]. The disease burden is immense and complications are common. One of the most common and serious complications is cardiovascular disease (CVD), which affects more than 30% of all patients with diabetes [3]. Other complications include retinopathy, neuropathy, and nephropathy.

Chronic kidney disease (CKD) affects up to 50% of all patients with type 2 diabetes mellitus (DM2) [4,5] and is common in patients without diabetes as well. It ranks just below diabetes as the 10th leading global cause of death, resulting in an estimated 1.3 million deaths annually. The most common cause of death in both patients with diabetes and patients with CKD is CVD [3,6].

Recently, sodium-glucose-cotransporter 2 (SGLT) inhibitors (SGLT2is) have revolutionized the treatment of both DM2 and CKD. Originally developed as an antidiabetic medication, SGLT2is block the SGLT2 channels in the proximal kidney tubule, inhibiting the reabsorption of sodium and glucose from the preurine, leading to glycosuria and a modest decrease in plasma glucose [7].

Several recent large randomized controlled clinical trials (RCTs) have shown that SGLT2is exert remarkable effects in patients with diabetes, both with and without CKD, reducing the risk of death by CVD, hospitalization for heart failure, and progression of CKD [8-10]. The effects are similar in patients with nondiabetic CKD, reducing the risk of CKD progression and death by renal or cardiovascular causes by 30% to 40% [11,12]. As a consequence, current guidelines recommend treatment with SLGT2i for patients with DM2 both with and without concomitant CKD, as well as for patients with nondiabetic CKD [13,14].

The mechanisms underlying these remarkable effects are, however, not yet fully understood. There are several possible pathways [15]. In this study, we examine 4 pathways.

### SLGT2i and Renal Hemodynamics

Increased renal reabsorption of sodium and glucose mediated by the SGLT2 channels is thought to be an important pathophysiological feature of diabetic nephropathy, instigating an increase in renal blood flow (RBF) via tubule-glomerular feedback mechanisms [16]. This, in turn, causes renal hyperfiltration; intraglomerular hypertension; and in time, kidney damage.

Intraglomerular hypertension is not unique to diabetic nephropathy but is thought to play a key role in the pathophysiology of nondiabetic CKD as well. A decline in functioning nephrons leads to a cascade of maladaptive hemodynamic changes, including increased intraglomerular pressure and hyperfiltration in the remaining nephrons [17,18].

Conversely, by blocking the SLGT2 channels, SGLT2is are hypothesized to alleviate the changes by reducing RBF and hyperfiltration, which could be a possible explanation of the observed beneficial effects. The acute drop in estimated glomerular filtration rate (eGFR) seen after initiation of SGLT2is could be a part of this mechanism as well [19]. While the effects of eGFR are well known, the reduction in RBF has only been demonstrated in a single study in patients with type 1 diabetes as well as in animal models [20,21]. None of the studies examining hemodynamic effects of SGLT2is in DM2 have found decreases in RBF [22,23], and it has, to our knowledge, never been examined in patients with CKD.

### SGLT2i and Sodium Balance

In addition to blocking glucose reuptake, SGLT2is inhibit sodium reuptake in the proximal tubule, which leads to a transient increase in urinary sodium excretion [24]. Furthermore, a modest reduction in body weight, plasma volume, and a decrease in sodium skin content is seen and may point to a decrease in volume status and total body sodium [25,26]. This might help explain the rapid improvement in cardiac function seen after SGLT2i initiation [27]. However, the natriuretic effects of SGLT2is seem to dissipate quickly, possibly due to renal compensatory mechanisms [28]. These mechanisms are not yet fully understood and have been sparsely studied.

### SGLT2i and Vascular Function

SGLT2is are also thought to exert an effect on the endothelial cells lining the inner walls of the blood vessels. DM2, CKD, and especially the combination of the 2 are associated with endothelial dysfunction and the subtle signs of endothelial dysfunction are often evident long before the clinical signs of vascular damage [29,30]. One of the most important reasons for endothelial dysfunction is the decreased synthesis and bioavailability of nitric oxide (NO), which leads to a decrease in systemic vasodilation, dysfunctional cell adhesion, smooth muscle cell proliferation, and hypercoagulability [31,32]. SGLT2is improve endothelial function in animal studies and seems to be able to improve vascular function and arterial stiffness in patients with DM2 [33-36]. So far, no studies have examined the effects in patients with CKD.

### SGLT2i and the Immune System

Inflammatory changes are common in the kidneys of patients with CKD and could be an important component in the development of glomerular injury, albuminuria, and CKD progression [37]. The innate immune system is involved in CKD progression and could be a target for SGLT2is. Both pattern recognition molecules (eg, collectin and mannan-binding lectin [MBL]) and complement activation pathways, notably the lectin pathway, could be involved in the progression of CKD, particularly in diabetes. Therefore, it is of interest to examine this system in diabetic and nondiabetic CKD and determine sensitivity to SGLT2is. Complement system components such as collectins, split products such as anaphylatoxins C3a and C5a, and terminal complexes (membrane attack complex) can be measured in plasma and urine along with other markers of the immune system [38,39]. SGLT2is can reduce the expression of inflammatory molecules such as tumor necrosis factor-α (TNF-α) and interleukin 6 (IL-6) [40]. Whether SGLT2is also affect markers of the immune system in the kidney remains to be examined. Since the complement system is associated with cell surfaces, we plan to use urine microvesicles (exosomes) and use them as imprints or “wet biopsies” for apical membrane deposition of complement activation products along with quantitation of soluble components in plasma and urine.

### Aims and Hypotheses

We aim to examine the effects of the SGLT2i empagliflozin versus placebo on renal hemodynamics, vascular function, sodium balance, and markers of the immune system in patients with DM2 with and without CKD, as well as in patients with nondiabetic CKD, hereby reflecting patient populations who would be offered SGLT2is in a clinical setting. In this paper, we describe and discuss our research hypothesis and the methods we used.

We hypothesize that the SGLT2i results in the following changes: (1) SLGT2i reduces RBF and glomerular filtration rate (GFR); (2) SGLT2i increases NO activity and improves endothelial function; (3) SGLT2i increases fractional sodium excretion, which is modified by more distally localized compensatory mechanisms; (4) SGLT2i increases renin angiotensin aldosterone system activity and decreases 24-hour ambulatory blood pressure and arterial stiffness; and (5) SLGT2i decreases renal innate immune activity.

## Methods

### Design

We conducted 3 double-blind, crossover RCTs, each with identical study protocols but different study populations. We included patients with (1) DM2 and preserved kidney function, (2) DM2 and CKD, and (3) nondiabetic CKD.

Each participant started with a run-in period of at least 2 weeks, wherein ongoing SGLT2i or nonsteroidal anti-inflammatory drug (NSAID) treatment, which is known to affect both renal hemodynamics and fluid homeostasis [41], was paused. If patients were not treated with SGLT2i or NSAID prior to inclusion, they could proceed directly to randomization. If deemed necessary, the SLGT2i could be substituted with a different class of antidiabetic treatment. The substitution was done in accordance with national treatment guidelines at the time [42]. After run-in, participants were randomly assigned to 4 weeks of treatment with either 10 mg of empagliflozin or a matching placebo, both taken once daily. After a wash-out period of at least 2 weeks, participants were crossed over to 4 weeks of the opposite treatment. Each 4-week treatment period was finalized with 2 examination days: 1 day at The University Clinic in Nephrology and Hypertension, Gødstrup Hospital, Denmark, and 1 day at The Department of Renal Medicine, Aarhus University Hospital, Denmark.

**Figure 1:**
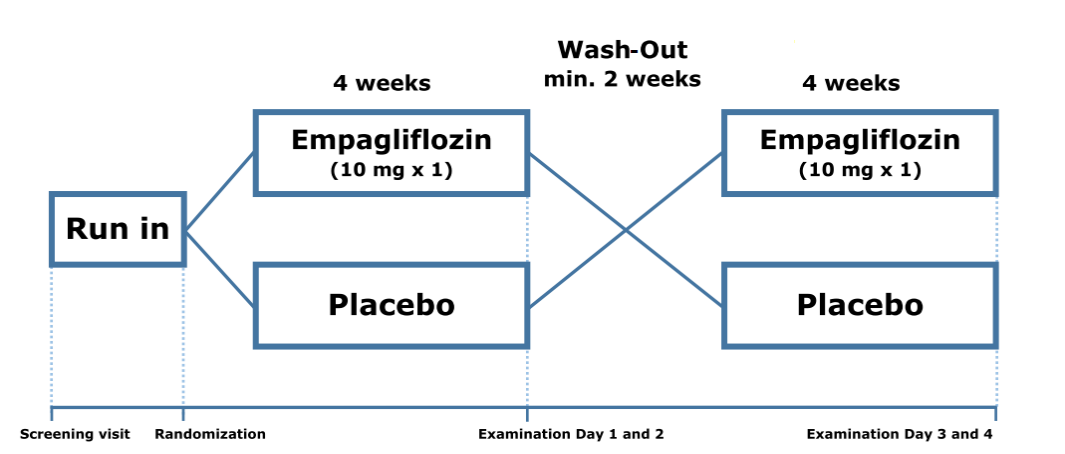
Study design.

We aimed to keep examination days adjacent, but due to logistic considerations, we allowed for an interval of up to 1 week between examination days in each treatment period. If there was an interval between the examination days, the treatment period was extended concomitantly, ensuring that the last dose of study medication was taken on the morning of the last examination day (Figure 1).

### Recruitment and Screening

Participants were recruited through announcements at general practitioners, through newspaper advertisements, and through email or letters to participants from previous trials, who have consented to being contacted about new trials. Furthermore, participants could be recruited from the outpatient clinics at The Department of Internal Medicine, Nephrology and The Department of Internal Medicine, Endocrinology, Gødstrup Hospital, Denmark.

Participants were screened prior to inclusion to ensure they fulfilled all inclusion criteria and none of the exclusion criteria. Screening involved physical examination; medical history; office blood pressure; electrocardiogram; urine test strip measurement; urinary albumin and albumin-to-creatinine ratio; and the following blood samples: p-glucose, p-alanine transaminase, b-glycated hemoglobin A_1c_ (HbA_1c_), p-potassium, p-sodium, p-albumin, p-creatinine, eGFR, B-leukocytes, B-hemoglobin, B-erythrocytes, and B-thrombocytes. Office blood pressure was measured 3 times and calculated as an average of the last 2 measurements. It was measured in both arms, and in case of difference between arms, the arm with the highest values was chosen for further measurements. If identical, the left arm was chosen. Inclusion, exclusion, and withdrawal criteria can be seen in Textbox 1.

**Inclusion, exclusion, and withdrawal criteria.**

**Inclusion criteria**

Study 1: Type 2 diabetes mellitus (DM2) and preserved kidney function

- Aged 18 years or older
- Estimated glomerular filtration rate (eGFR) > 60 mL/min/1.73 m^2^
- DM2 was diagnosed at least 1 year before inclusion and in stable medical antidiabetic treatment for at least 3 months
- Hemoglobin A_1c_ (HbA1c) 48-70 mmol/mol (Diabetes Control and Complications Trial [DCCT] values 6.5%-8.6%)
- Fertile women were to use safe contraception

Study 2: DM2 and chronic kidney disease (CKD)

- Aged 18 years or older
- eGFR 20-60 mL/min/1.73 m^2^
- DM2 was diagnosed at least 1 year before inclusion, and in stable medical antidiabetic treatment for at least 3 months
- HbA_1c_ 48-70 mmol/mol. (DCCT values 6.5%-8.6%)
- Fertile women were to use safe contraception

Study 3: Nondiabetic CKD

- Aged 18 years or older
- eGFR 20-60 mL/min/1.73 m^2^
- Fertile women were to use safe contraception

**Exclusion criteria**

Study 1 and 2: DM2 and preserved kidney function and DM2 and CKD

- Type 1 diabetes
- Alcohol or substance abuse
- Pregnancy or breastfeeding
- Anamnestic or clinical signs of heart or liver failure
- Active cancers, aside from skin cancers (spinocellular or basocellular carcinomas)
- BMI > 35 kg/m^2^
- Allergies or unacceptable side effects to the experimental treatment or background treatment
- If the investigator found the participant unfit to complete the trial.
- Previous kidney transplant
- Autosomal dominant polycystic kidney disease (ADPKD).

Study 3: Nondiabetic CKD

- Same as in study 1 and study 2
- DM2

**Withdrawal criteria**

- For all 3 studies, participants were withdrawn if they developed an exclusion criterion, withdrew consent, were noncompliant, or experienced serious or unacceptable adverse events.

### Participants

Our power calculation was based on the primary end point, RBF. In total, 15 patients were needed in each study to detect a minimal relevant difference in RBF of 0.167 mL/min/g, with an SD of 0.180 mL/min/g, a 2-sided α-level of 5%, and a power of 90%.

### Randomization and Study End Points

Randomization numbers were provided by the manufacturer, Boehringer-Ingelheim. Randomization was performed by the Hospital Pharmacy, Central Denmark Region, Department Gødstrup. Participants were randomized in blocks of 4. Treatment assignment and allocation were concealed from clinicians, participants, and research staff until the trials were completed and as long as they were involved in data analysis. A copy of the randomization list and sealed envelopes with individual randomization numbers were kept in a locked safe at The University Clinic of Nephrology and Hypertension, in case unblinding was required. A single participant could be unblinded without it affecting the rest of the trial. At the end of the project, the envelopes will be returned to the Hospital Pharmacy where a receipt will be drawn up. The primary and secondary study end points can be viewed in Textbox 2.

**Primary and secondary study end points.**

**Primary end point**

- Renal blood flow

**Secondary end points**

- Glomerular filtration rate
- Renal vascular resistance (RVR), filtration fraction, afferent and efferent arteriolar resistance (Ra and Re)
- Vascular function
- The nitric oxide–system, measured as plasma and urinary levels of nitrite, nitrate, and cyclic guanosine monophosphate (cGMP)
- The complement system, measured as plasma and urine levels of mannan-binding lectin (MBL), collectin kidney 1 (CL-K1), collectin liver 1 (CL-L1), mannan-binding lectin serine protease (MASP) 1-3, C4c, C3c, C3dg, sC5b-9, and urinary exosomes.
- Systemic hemodynamics, measured as 24-hour ambulatory brachial blood pressure, heart rate, pulse wave velocity, and peripheral resistance
- Plasma levels of renin, angiotensin II, aldosterone, vasopressin, and brain natriuretic peptide
- Water and sodium excretion: urinary sodium, free water clearance, urinary glucose, urinary albumin, fractional sodium excretion, urinary excretion of tubular transporter proteins (aquaporin 2 [AQP2], endothelial sodium channel [ENaC], sodium chloride symporter channel [NCC], and sodium-potassium-chloride cotransporter [NKCC]), extracellular body water (EBW), total body water, intracellular body water (IBW), adipose tissue mass, and erythrocyte salt sensitivity
- Hemoglobin A_1c_ (HbA_1c_) and p-glucose
- β-hydroxy butyrate and urate
- Urinary excretion of adenosine, neutrophil gelatinase-associated lipocalin (NGAL), kidney injury molecule-1 (KIM-1), and interleukin 6 (IL-6)
- Plasma concentrations of parathyroid hormone, phosphate, calcium, alkali phosphatase and fibroblast growth factor (FGF23), and urinary excretion of phosphate and calcium

### Study Medication

The active treatment, 10 mg of empagliflozin, and a matching placebo were produced and distributed by the manufacturer, Boehringer-Ingelheim. The placebo tablet was identical to empagliflozin in every way, except for the lack of active substance. The study medication was delivered from The Hospital Pharmacy in identical pill bottles and was allocated corresponding to the randomization number.

Compliance was checked with a phone call midway through each examination period and by pill count when the study medication was returned on the last examination day.

### Study Methods

#### ^82^Rubidium Positron Emission Tomography–Computed Tomography

RBF was measured with ^82^Rubidium positron emission tomography–computed tomography (^82^Rb-PET/CT) scans. All scans were performed on a Siemens Biograph mCT; 64 slice-4R (Siemens Healthcare GmbH). The method has been previously described by Langaa et al [43,44].

Participants rested in a sitting position for at least 30 minutes prior to the scan. During the scan, participants were placed in a supine position with arms resting over their heads. After positioning, a low-dose CT scan (25 mAs, 100 kV) was performed for attenuation control. This was immediately followed by a bolus injection of 555 megabecquerel (MBq) ^82^Rb through a peripheral venous catheter (PVC), whereafter an 8-minute dynamic PET scan was performed. Through iterative reconstruction, 3D images of the activity changes in the abdominal aorta and the parenchyma of both kidneys were generated.

A 1-tissue compartment model was used for flow estimation, and RBF was calculated as a K1-value based on activity uptake in the abdominal aorta and both kidneys. Values were calculated using PMOD (PMOD Technologies Ltd).

^82^Rb was obtained using an ^82^Rb-generator (Cardiogen-82; Bracco Diagnostics Inc). Scans were done in cooperation with The Department of Nuclear Medicine, Regional Hospital Gødstrup.

#### Venous Occlusion Strain Gauge Plethysmography

Vasodilatory function was measured using classic forearm venous occlusion plethysmography (Hokanson EC6) as previously described by Fredslund et al [45], although we did not perform measurements in the contralateral arm. An indium-gallium strain gauge placed around the forearm senses changes in forearm volume. Changes in forearm volume during brief, very fast, occlusions (using the Hokanson E20 inflator) of venous outflow by a cuff on the upper arm will then reflect the arterial inflow. The plethysmography method, therefore, allows direct assessment of the effects of vasoactive drugs infused into the brachial artery. In this project, we used the infusion of acetylcholine (ACh) and sodium nitroprusside (SNP) for the evaluation of endothelium-dependent and independent vasodilatation, respectively.

#### GFR

GFR was determined through clearance of ^99m^Technetium-diethylene-triamine-pentaacetate (^99m^Tc-DTPA). Through a PVC, 25 MBq of ^99m^Tc-DTPA was injected intravenously. Before injection, a zero sample was drawn. Blood samples were drawn after 3, 4, and 5 hours, measuring residual plasma ^99m^Tc-DTPA activity, whereby GFR was calculated. Measurements were done in cooperation with The Department of Nuclear Medicine, Regional Hospital Gødstrup.

#### Blood Pressure Measurement and Arterial Stiffness

24-Hour ambulatory brachial blood pressure, heart rate, pulse wave velocity, and arterial stiffness were measured with Mobil-O-Graph (IEM GmbH).

#### Biochemical Analyses

Plasma and serum levels of sodium, HbA_1c_, glucose, brain natriuretic peptide, potassium, albumin, creatinine, phosphate, parathyroid hormone, alkali phosphatase, urate, total protein, hemoglobin, erythrocyte volume fraction, thrombocytes, and calcium were routinely analyzed by The Department of Biochemistry, Gødstrup Hospital, Denmark. β-hydroxy butyrate was measured with a FreeStyle Precision Neo point of care devise (Abbott Laboratories).

Plasma levels of renin, angiotensin II, aldosterone, and vasopressin were measured by radioimmunoassay. Plasma levels of cyclic guanosine monophosphate (cGMP) and fibroblast growth factor 23 (FGF23) were measured by enzyme-linked immunosorbent assay (ELISA).

Plasma and urine levels of nitrite and nitrate were measured by spectrophotometry.

Erythrocyte salt sensitivity was measured with a salt blood test (CARE Diagnostica Laborreagenzien GmbH). Plasma and urinary osmolality were measured by freeze point depression with an A2O osmometer (Advanced Instruments).

Plasma and urinary levels of MBL, collectin kidney 1 (CL-K1), collectin liver 1 (CL-L1), mannan-binding lectin serine protease (MASP) 1-3, C4c, C3c, C3dg, sC5b-9, and urinary exosomes were measured at The Department of Cardiovascular and Renal Research, University of Southern Denmark.

Urine volume and urinary levels of sodium, creatinine, albumin, calcium, and phosphate were measured routinely by The Department of Biochemistry, Gødstrup Hospital, Denmark.

Urinary levels of glucose, aquaporin 2 (AQP2), epithelial sodium channel (ENaC), sodium chloride symporter channel (NCC), and sodium-potassium-chloride cotransporter (NKCC) were measured by radioimmunoassay; cGMP, IL6, neutrophil gelatinase-associated lipocalin (NGAL), kidney injury molecule-1 (KIM-1), and adenosine were measured by ELISA.

#### Bioimpedance Measurement

EBW, total body water, intracellular body water, and Adipose Tissue Mass were measured with bioimpedance spectroscopy using a body composition monitor (Fresenius Medical Care AG & Co KGaA).

#### Renal Hemodynamics

Renal vascular resistance was calculated as mean arterial pressure/RBF. Filtration fraction was calculated as GFR/renal plasma flow. Afferent and efferent arteriolar resistance were calculated using the Gomez equations [46].

### Statistics

Data following a normal distribution was calculated with parametric statistics. Paired data were compared with either paired 2-tailed *t* test or ANOVA. Nonparametric statistics were applied if variables were not normally disrupted. Paired comparisons were compared with Wilcoxon Signed Rank test or Friedman test. Statistics were performed using STATA (version 18.0; StataCorp LLC).

### Experimental Procedure

#### Prior to Examination

Fluid intake was standardized from 4 days prior to the first examination day till the last examination day in each treatment period. Each participant was encouraged to drink at least 2 L of water per day, and 2 cups of coffee or tea daily was allowed, except for 8 hours prior to the examinations. Alcohol or soft drinks were prohibited in this period. The use of mouthwash products was prohibited throughout the study period. Furthermore, in each treatment period, participants were encouraged to adhere to their usual diets from 4 days prior to the first examination and till the last examination. Participants were not required to fast prior to the examination but did not eat throughout the examinations.

#### Examination Day: University Clinic in Nephrology and Hypertension, Gødstrup Hospital

**Figure 2:**
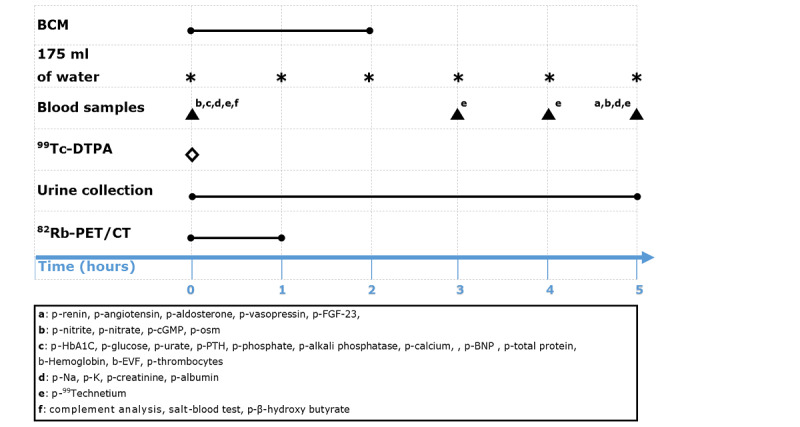
Schematic illustration of the examination day at Gødstrup Hospital. 82Rb-PET/CT: ^82^Rubidium positron emission tomography/ computed tomography; ^99m^Technetium-diethylene-triamine-pentaacetate (^99m^Tc-DTPA); BCM: body composition monitor.

Participants were set to meet at 8 AM, having ingested their usual medication and the study medication. First, participants emptied their bladder, body weight was measured, and they rested in a chair for 30 minutes. A pregnancy test was performed on fertile women. Then, a PVC was inserted and the Mobil-O-Graph was placed on the upper arm, opposite the PVC. At time point 0 blood samples were drawn and a bolus injection of ^99m^Tc-DTPA was given (Figure 2). After this, the ^82^Rb-PET/CT scan was performed. Two successive body composition monitor measurements were done within the first 2 hours of the examination day.

Urine was collected at 2 and 5 hours and if additional voiding was necessary. All urine, except the first void, was pooled and analyzed. From the first void, 50 mL of fresh spot urine was collected, a protease inhibitor was added, and the sample was frozen for later exosome analyses. Two mL of spot urine, also from the first void, was frozen for analysis of complement factors. Blood samples were drawn at 3, 4, and 5 hours. At the end of the examination day, body weight was measured again. The Mobil-O-Graph was removed by the participant 24 hours after mounting. During the entire examination day, participants were to rest in a bed or on a chair. Voiding was done standing or sitting. Participants were given 175 mL of water each hour from time point 0 till the end of the examination day.

#### Examination Day: Department of Renal Medicine, Aarhus University Hospital

**Figure 3:**
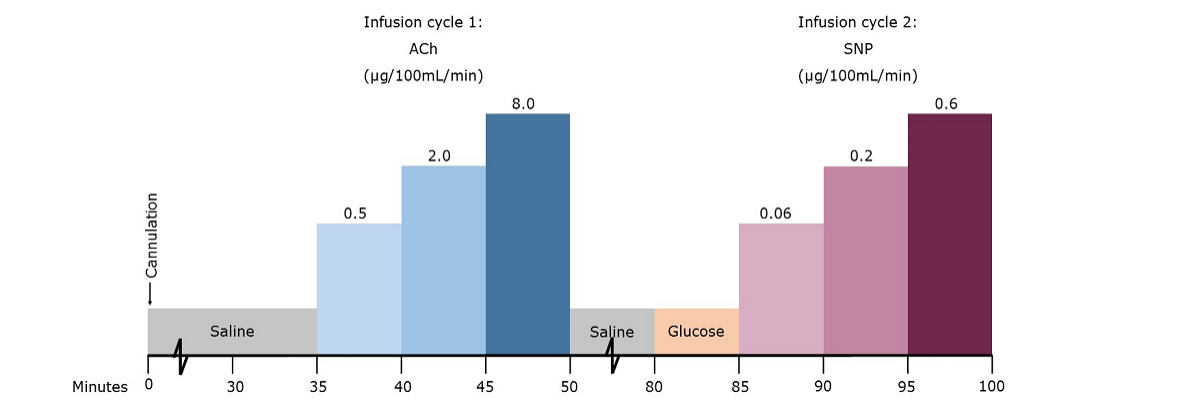
Schematic timetable of the strain gauge plethysmography experimental protocol using ACh. ACh: acetylcholine; SNP: sodium nitroprusside.

**Figure 4:**
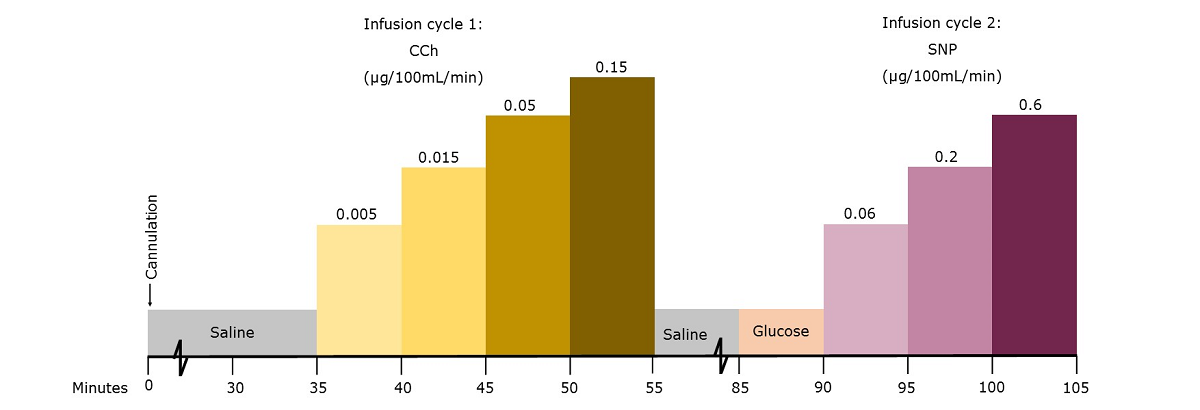
Schematic timetable of the strain gauge plethysmography experimental protocol using CCh. CCh: carbachol; SNP: sodium nitroprusside.

The strain gauge plethysmography was performed at the Department of Renal Medicine, Aarhus University Hospital (Figures 3 and 4). Participants had taken their usual medication and the study medication on the morning prior to the examination. Participants were placed in a supine position in a room where the temperature was kept fixed at 25 °C. The brachial artery was then cannulated with a 27-gauge needle and kept from clotting by a slow infusion of isotonic saline. The strain gauge was placed on the broadest part of the forearm, a venous occlusion cuff was placed on the upper arm, and an arterial occlusion cuff was placed at the wrist. After 30 minutes of saline infusion, baseline measurements were recorded (8 readings were performed per measurement). ACh was infused in increasing concentrations at 5-minute intervals. Measurements were recorded at each concentration. After the last measurement, saline was infused for another 30 minutes. Afterward, isotonic glucose was infused for 5 minutes, and new baseline measurements were recorded. SNP was now infused in increasing concentrations again at 5-minute intervals, with measurements done at each concentration. Arterial circulation to the hand was interrupted during infusion. When the last measurement had been performed, the infusion was stopped, the needle was removed, and the examination day ended.

Due to delivery issues, ACh was replaced with carbachol (CCh) after the first 23 participants had been examined. Two participants, both in the DM2 and CKD group, had their first examination done with ACh and the second with CCh. The remaining examinations were done entirely with CCh.

### Ethical Considerations

All 3 studies were approved by The Central Denmark Region Committees on Health Research Ethics (cases: 1-10-72-214-20, 1-10-72-339-20, and 1-10-72-340-20, respectively) and The Danish Medicines Agency (EU Clinical Trials Register: 2019-004303-12, 2019-004447-80, and 2019-004467-50) and were conducted in accordance with the Declaration of Helsinki 2013. Informed and signed consent was obtained from all participants. The study was monitored by the Good Clinical Practice (GCP) Unit of Aarhus and Aalborg University Hospitals. All the data have been deidentified. Participants could be compensated for travel expenses related to the studies.

## Results

Approval from the regulatory agencies was obtained for the first study (DM2 and preserved kidney function) by October 2020. Recruitment began in April 2021. Approval for the remaining studies was obtained by May 2021 and recruitment began in March 2022. The inclusion of participants for all studies was completed by September 2022. Examinations started in August 2021 and were completed (last patient, last visit) by December 2022. In total, 49 participants completed the project: 16 in the DM2 and preserved kidney function study, 17 in the DM2 and CKD study, and 16 in the nondiabetic CKD study. Data analysis is currently ongoing. So far, no results from the project have been published.

## Discussion

### Future Perspectives

In this paper, we present the background, hypothesis, design, and methodology of a project consisting of 3 RCTs examining the effects of the SGLT2i empagliflozin versus placebo on a wide range of parameters in different patient populations. This will hopefully provide important information on the mechanisms underlying the beneficial effects of SGLT2is and is the first project to examine both patients with and without diabetes and with and without CKD using the same experimental setup. To our knowledge, very few mechanistic studies have examined SGLT2is in a CKD population.

We use a novel method for estimating RBF by using ^82^Rb-PET/CT. Compared with measuring effective renal plasma flow (ERPF) with para-aminohippuric acid (PAH) clearance, a method often used in other studies examining hemodynamic effects of SGLT2is [20,23,47], flow estimation with ^82^Rb-PET/CT can be performed much quicker (in less than half an hour) without the need for blood or urine samples and allows for estimation of single kidney RBF. Furthermore, the effective radiation dose of a single scan is limited (≈1 milliSievert [mSv]). There is an ongoing discussion around whether our method in fact reflects RBF or rather RPF. This should be taken into account when calculating intrarenal hemodynamics since the calculations rely on which parameter is used. However, the method has proven both precise and reliable when evaluating relative changes in kidney perfusion, which is what we assess in this project [43,44].

Vascular function and SGLT2is have been studied widely, as specified in the introduction, though mainly by flow-mediated dilation [34,35]. The venous occlusion strain-gauge plethysmography gives a more in-depth examination of the potential mechanisms at play by measuring both endothelial-dependent and independent vasodilation.

A clear strength of our project is the robust, randomized, placebo-controlled, crossover design. We examine different patient populations using the exact same design, allowing for comparison of effect between groups. Furthermore, we investigate a number of different variables, allowing for different mechanisms to be examined. With the current updated guidelines, all 3 examined groups represent patient populations who would be offered SGLT2is in a clinical setting, which adds to the generalizability of our results. It is important to note that inclusion in the DM2 and CKD group did not require a diagnosis of diabetic nephropathy, so participants could potentially have kidney disease or other etiologies. We used 10 mg of empagliflozin and not the 25-mg dose, since this was the dose used in the EMPA-KIDNEY study and since both doses have equivalent effects on both renal outcomes and eGFR decline [8].

### Limitations

Our project has several limitations: one being the small study sample sizes, increasing the risk of the studies being underpowered for detecting changes in secondary end points. In addition to being a strength, examining multiple mechanisms of action can be a limitation, since it increases the risk of type 1 errors; thus, most of our secondary end points should be interpreted as exploratory. Our inclusion criteria are broad by design, mainly to reflect a real-world patient population that could potentially be prescribed SGLT2is, but the broad criteria make for a more heterogeneous study population and increase the risk of heterogeneity of the outcome effects. Despite being a well-known risk factor for disease progression in both DM2 and CKD [48], we did not make albuminuria an inclusion criterion. We chose this approach because albuminuria was not an inclusion criterion for all patients in either the EMPA-REG OUTCOME, DECLARE-TIMI 58, or EMPA-KIDNEY trial [49-51], although it was in the CREDENCE and DAPA-CKD trials [9,12]. By allowing the inclusion of patients without albuminuria, we risk including patients at low risk where treatment benefits could be less pronounced. Furthermore, patients were not fasting before examinations, nor did we take steps to ensure stable blood glucose levels throughout the examination days. This was considered, but discarded for feasibility reasons, but it does increase the risk of our outcomes being affected by confounders. Adding to this was the fact that we allowed for the substitution of antidiabetic treatment in the run-in period if SGLT2is were paused. This was done to ensure glycemic control during the trial period but may have introduced further confounding. This methods paper was written after data collection was completed. While it would have been optimal to publish it prior to inclusion, we judge this to be a precise description of the methods used and it will provide a valuable framework for future articles, detailing exactly how our results were obtained and what considerations lay behind them.

### Conclusions

This paper describes the rationale, design, and method used in a project consisting of 3 double-blind, crossover RCTs, examining the effects of empagliflozin versus placebo in patients with DM2 with and without CKD and patients with nondiabetic CKD, respectively.

## Abbreviations

^82^Rb: ^82^Rubidium
^99m^Tc: ^99m^Technetium
ACh: acetylcholine
AQP2: aquaporin 2
CCh: carbachol
cGMP: cyclic guanosine monophosphate
CKD: chronic kidney disease
CL-K1: collectin kidney 1
CL-L1: collectin liver 1
CT: computer tomography
CVD: cardiovascular disease
DM2: type 2 diabetes mellitus
DTPA: diethylene-triamine-pentaacetate
EBW: extracellular body water
eGFR: estimated glomerular filtration rate
ELISA: enzyme-linked immunosorbent assay
ENaC: epithelial sodium channel
ERPF: effective renal plasma flow
FGF23: fibroblast growth factor 23
GFR: glomerular filtration rate
HbA_1c_: hemoglobin A_1c_
IL-6: interleukin 6
KIM-1: kidney injury molecule-1
MASP: mannan-binding lectin serine protease
MBL: mannan-binding lectin
MBq: megabecquerel
mSv: milliSievert
NCC: sodium chloride symporter channel
NGAL: neutrophil gelatinase-associated lipocalin
NKCC: sodium-potassium-chloride cotransporter
NO: nitric oxide
NSAID: nonsteroidal anti-inflammatory drugs
PAH: para-aminohippuric acid
PET: positron emission tomography
PVC: peripheral venous catheter
PWV: pulse wave velocity
RBF: renal blood flow
RCT: randomized controlled trial
RVR: renal vascular resistance
SGLT2: sodium-glucose-cotransporter 2
SGLT2i: sodium-glucose-cotransporter 2 inhibitor
SNP: sodium nitroprusside
TNF-α: tumor necrosis factor-α
